# BTLA/HVEM Axis Induces NK Cell Immunosuppression and Poor Outcome in Chronic Lymphocytic Leukemia

**DOI:** 10.3390/cancers13081766

**Published:** 2021-04-07

**Authors:** Christian Sordo-Bahamonde, Seila Lorenzo-Herrero, Ana P Gonzalez-Rodriguez, Ángel R. Payer, Esther González-García, Alejandro López-Soto, Segundo Gonzalez

**Affiliations:** 1 Department of Functional Biology, Immunology, Universidad de Oviedo, 33006 Oviedo, Spain; Christiansbl87@gmail.com (C.S.-B.); seilalorenzoherrero@gmail.com (S.L.-H.); 2 Instituto Universitario de Oncología del Principado de Asturias (IUOPA), 33006 Oviedo, Spain; apayer.angel@gmail.com (Á.R.P.); lopezsalejandro@uniovi.es (A.L.-S.); 3 Instituto de Investigación Sanitaria del Principado de Asturias (ISPA), 33011 Oviedo, Spain; 4 Department of Hematology, Hospital Universitario Central de Asturias (HUCA), 33011 Oviedo, Spain; 5 Department of Hematology, Hospital de Cabueñes, 33203 Gijón, Spain; esthergongar@yahoo.es; 6 Department of Biochemistry and Molecular Biology, University of Oviedo, 33006 Oviedo, Spain

**Keywords:** Chronic lymphocytic leukemia, CLL, BTLA, HVEM, TNFRSF14, CD270, CD272, NK cell, immunotherapy, checkpoint

## Abstract

**Simple Summary:**

Chronic lymphocytic leukemia (CLL) represents the most frequent B cell malignancy in Western countries and still remains as an incurable disease. Despite recent advances in targeted therapies including ibrutinib, idelalisib or venetoclax, resistance mechanisms have been described and patients develop a progressive immunosuppression. Since immune checkpoint blockade has demonstrated to reinvigorate T and NK cell-mediated anti-tumor responses, the aim of this work was to elucidate whether this immunosuppression relies, at least in part, in BTLA/HVEM axis in patients with CLL. Our results demonstrate that BTLA and HVEM expression is deeply dysregulated on leukemic and NK cells and correlates with poor outcome. Moreover, soluble BTLA levels correlated with adverse cytogenetics and shorter time to treatment. BTLA blockade restored, at least in part, NK cell-mediated responses in patients with CLL. Altogether, our results provide the rationale to further investigate the role of BTLA/HVEM axis in the pathogenesis of CLL.

**Abstract:**

Chronic lymphocytic leukemia (CLL) is characterized by progressive immunosuppression and diminished cancer immunosurveillance. Immune checkpoint blockade (ICB)-based therapies, a major breakthrough against cancer, have emerged as a powerful tool to reinvigorate antitumor responses. Herein, we analyzed the role of the novel inhibitory checkpoint BTLA and its ligand, HVEM, in the regulation of leukemic and natural killer (NK) cells in CLL. Flow cytometry analyses showed that BTLA expression is upregulated on leukemic cells and NK cells from patients with CLL, whereas HVEM is downregulated only in leukemic cells, especially in patients with advanced Rai-Binet stage. In silico analysis revealed that increased *HVEM*, but not *BTLA*, mRNA expression in leukemic cells correlated with diminished overall survival. Further, soluble BTLA (sBTLA) was found to be increased in the sera of patients with CLL and highly correlated with poor prognostic markers and shorter time to treatment. BTLA blockade with an anti-BTLA monoclonal antibody depleted leukemic cells and boosted NK cell-mediated responses ex vivo by increasing their IFN-γ production, cytotoxic capability, and antibody-dependent cytotoxicity (ADCC). In agreement with an inhibitory role of BTLA in NK cells, surface BTLA expression on NK cells was associated with poor outcome in patients with CLL. Overall, this study is the first to bring to light a role of BTLA/HVEM in the suppression of NK cell-mediated immune responses in CLL and its impact on patient’s prognosis, suggesting that BTLA/HVEM axis may be a potential therapeutic target in this disease.

## 1. Introduction

Chronic lymphocytic leukemia (CLL), the most prevalent adult leukemia in Western countries, still remains as an incurable disease. This malignancy is characterized by progressive clonal accumulation of mature B cells in peripheral blood, spleen, bone marrow and lymph nodes [1]. Clinically, patients with CLL show a vast outcome variability, with patients having an indolent curse of the disease, whereas others suffer from a life-threatening disease [2,3]. To date, the landscape of CLL treatment includes chemotherapy and anti-CD20 monoclonal antibodies (mAb) [4]. Within the last years, new therapeutic options have become available, including Bruton’s tyrosine kinase (BTK) inhibitor ibrutinib, phosphatyidilinositol-3 kinase inhibitor idelalisib and B-cell lymphoma 2 (BCL-2) antagonist venetoclax [5,6,7]. Despite the fact that these therapies have been effective in patients who relapsed after chemotherapy, resistance mechanisms have already been reported [8,9].

Patients with CLL acquire a progressive immune dysfunction affecting both adaptive and innate immune responses [10,11,12,13,14,15,16]. In this context, growing evidence suggests that immune checkpoint blockade (ICB)-based therapies, a major breakthrough in the fight against cancer, may reinvigorate immunosurveillance, hence being a therapeutic option for patients with CLL. Although most attention in ICB therapy has been focused on the role of T cells, there is an increasing interest in blocking inhibitory pathways dampening NK cell-mediated responses [17,18,19,20,21,22,23]. NK cells from patients with CLL show decreased expression of activating receptors and augmented expression of inhibitory receptors and checkpoint proteins, leading to NK cell dysfunction [10,24,25]. Thereby, diminished NK cell numbers and activity have been linked to poor outcome in CLL [24,26]. In addition, patients with CLL show increased risk of developing secondary neoplasia due to, at least in part, an attenuated immunosurveillance, thus highlighting the importance antitumor immunity in this disease [27,28,29]. Several studies reported the dysregulation of PD1/PD-L1 axis in CLL; however, even though preclinical data suggested that patients with CLL may take advantage from PD-1/PD-L1 blockade, no clinical benefit was achieved in clinical trials [30,31]. Nevertheless, our group and others have reported new potential checkpoint targets in CLL, such as CD200, ILT2, LAG-3 or NKG2A [25,32,33,34]. Thus, there is an increasing interest in developing new therapeutic strategies including targeting these novel immune checkpoints, alone or in combination with other therapies. In this line, treatment with relatlimab, a LAG-3 blocking mAb, in combination with nivolumab (anti-PD1 mAb) has demonstrated efficacy in vitro and in vivo and is currently being evaluated in clinical trials in different hematological malignancies, including CLL (NCT02061761) [35].

The immune checkpoint B- and T-lymphocyte attenuator (BTLA/CD272) and its ligand, Herpesvirus entry mediator (HVEM/CD270/TNFRSF14), are dysregulated in cancer. Importantly, HVEM functions as a bidirectional molecular switch between activating (CD160, LIGHT and lymphotoxin-α) and inhibitory (BTLA) pathways depending on the binding partner. Upon engagement, HVEM provides pro-survival and proliferative signals through activation of NF-kB transcriptional pathways, whereas BTLA negatively regulates T cell-mediated responses [36,37,38,39]. Further, aberrant surface expression and levels of soluble BTLA (sBTLA) in sera have been related to prognosis in various solid tumors and hematological malignancies [36,37,38,40,41,42,43,44,45,46,47]. BTLA blockade, alone or in combination with chemotherapy, led to improved survival in epithelial ovarian carcinoma murine models [48]. In melanoma, BTLA is highly expressed on tumor-specific CD8+ T cells and treatment with an anti-BTLA blocking mAb resulted in enhanced proliferative capability and cytokine production in vivo and in vitro [49]. Despite scarce data regarding the role of BTLA on NK cells being available, BTLA upregulation on NK cells was shown to compete with CD160, an activating binding partner for HVEM, to provide inhibitory signaling for NK cell cytotoxic activity, thus possibly impairing immunosurveillance [50]. In hematological malignancies, BTLA/HVEM axis dysregulation has been linked to T cell functional exhaustion and poor prognosis in diffuse large B cell lymphoma (DLBCL) and follicular lymphoma (FL) [42,51]. Moreover, CD160 is aberrantly overexpressed on leukemic cells from patients with CLL and other B-cell malignancies, thus promoting pro-survival and proliferative signaling to tumor cells [52,53,54]. Regarding CLL, there are only a few studies reporting BTLA and HVEM expression in patients, but nothing is known about the functional and clinical consequences in the antitumor immune response [55,56,57]. In fact, the potential impact of BTLA dysregulation in the modulation of T cell and NK cell-mediated responses has not yet fully been elucidated in neither hematological malignancies nor solid tumors.

Herein, we report a novel mechanism of immune suppression in CLL involving the dysregulation of BTLA/HVEM axis. Increased HVEM surface expression and high levels of sBTLA correlated with decreased overall survival (OS) and time to treatment (TTT) suggesting a role of BTLA/HVEM in the pathogenesis of CLL. mAb-mediated BTLA blockade (Genentech, San Francisco, CA, USA) enhanced NK cell-mediated responses, including IFN-γ production, cytotoxicity, and antibody-dependent cytotoxicity (ADCC), suggesting an immunosuppressive role for BTLA/HVEM in this disease. Altogether, our data support the rationale to further investigate the potential of BTLA blockade as a novel therapeutic strategy in CLL. 

## 2. Materials and Methods

### 2.1. Patients’ Samples

Blood samples from 46 consecutive non-treated patients with CLL were obtained from Hospital Universitario Central de Asturias and Hospital de Cabueñes (Table 1). All clinical analyses were performed according to International workshop on CLL guidelines criteria [58]. Written informed consent following the Declaration of Helsinki was obtained from all the patients and sample collection was performed with approval from the local ethics committee (Comité de Ética de la Investigación del Principado de Asturias, case-19042016). Patients with CLL were diagnosed according to clinical and laboratory criteria. Karyotype was categorized as unaltered, single abnormality or complex. FISH analysis for chromosome 13q deletion (del(13q)), 11q deletion (del11q)), 17p deletion (del(17p)) and trisomy 12 was performed. Positive patient cases were those with 5% or more cells with the abnormality. Variable region of the immunoglobulin heavy chain (*IGHV*) mutation status was characterized by direct sequencing method, and patients were categorized as unmutated (*IGHV* 98% germline homology) or mutated (98% homology). A total of 17 patients required treatment after sample collection (progressive disease) and 29 patients showed stable disease; therefore, not requiring further clinical intervention. The median follow-up from diagnosis of patients was 66 months. Samples from healthy donors (HD) were provided by Centro Comunitario de Sangre y Tejidos de Asturias. Peripheral blood mononuclear cells (PBMCs) from patients and HD were isolated by Ficoll density gradient centrifugation (Histopaque®-1077, Sigma-Aldrich, San Luis, MO, USA) and used fresh. PBMCs were cultured in RPMI 1640 (Lonza, Basilea, Switzerland) supplemented with 10% heat-inactivated fetal bovine serum (Sigma-Aldrich), 1 mM sodium pyruvate, 2 mM L-glutamine, 100 U/mL penicillin and 10 μg/mL streptomycin at 37 °C and 5% CO_2_. CLL-derived cell line MEC-1 was obtained from ATCC and cultured in Iscove’s Modified Dulbecco’s Medium (IMDM), supplemented as described above. 

### 2.2. Analysis of BTLA and HVEM Expression on Immune Cells

Leukemic cells, B lymphocytes and NK cells from patients and HD were identified by flow cytometry. For this purpose, the following antibodies were employed: anti-CD19-APC and anti-CD3-PE (Immunostep, Salamanca, Spain), anti-CD3-FITC and anti-CD56-APC (both from Cytognos, Salamanca, Spain). Leukemic cells were identified as CD19+. NK cells were defined as CD56+CD3−. BTLA and HVEM expression was evaluated using anti-BTLA-PE (clone MIH26, Biolegend, San Diego, CA, USA) and anti-HVEM-PE (clone 122, Biolegend). Cells were analyzed in a Cytoflex S flow cytometer and CytExpert 2.3 software (Beckman Coulter, Brea, CA, USA). 

### 2.3. Leukemic Cell Depletion Assay 

PBMCs from patients with CLL were freshly treated with anti-BTLA blocking antibody at 10 µg/mL (clone 3B1, kindly provided by Genentech, San Francisco, CA, USA) or isotype-matched control (kindly provided by Juan Ramón de los Toyos-González, Universidad de Oviedo, Oviedo, Spain) for indicated times. In order to evaluate the absolute leukemic cell count, PMBCs were stained for immune subset identification as described and an equal volume of PKH26 reference microbeads (Sigma-Aldrich, Sigma-Aldrich, San Luis, MO, USA) was added to each condition. A total of 5 × 10^3^ reference microbeads were acquired and analyzed by flow cytometry and the absolute number of leukemic cells was calculated. Percentage of leukemic cell count was normalized considering the control as 100%.

### 2.4. Intracellular Cytokine Staining

IFN-γ production by NK cells was evaluated as previously reported [59]. Briefly, PBMCs from patients with CLL were treated with anti-BTLA blocking antibody, control IgG for 72 h, plate-coated agonistic anti-BTLA antibody (clone MIH26, Biolegend, San Diego, CA, USA) or proper isotype control (all at 10 µg/mL) for 24 h. Afterwards, PBMCs from each condition were stimulated with 50 nM PMA and 1 µg/mL ionomycin for 4 h. After the first 1 h of incubation, brefeldin A (Biolegend) was added to avoid the release of cytokines to the cell culture media. Following incubation, cells were stained with anti-CD56-APC and anti-CD3-FITC to identify NK cells. For intracellular cytokine staining, BD Cytofix/Cytoperm Fixation/Permeabilization Kit (BD Biosciences, San Jose, CA, USA) was used according to the manufacturer’s instructions. NK cells were stained with anti-human IFN-γ-PE (clone 4S.B3, Biolegend) and IFN-γ+ NK cells were evaluated by flow cytometry. For IL-10 evaluation, PBMCs from patients with CLL were cultured unstimulated or stimulated with 200 ng/mL CpG (ODN2395, Miltenyi Biotec, Bergisch Gladbach, Germany) alone or in combination with blocking anti-BTLA mAb for 48 h. IL-10+CD19+ leukemic cells were determined as described above. 

### 2.5. Evaluation of NK Cell-Mediated Cytotoxicity

The effect of BTLA blockade on NK cell-mediated cytotoxicity and ADCC was assessed by calcein-AM staining as previously described by our group [60]. Total PBMCs from patients with CLL were treated with blocking anti-BTLA mAb or isotype-matched control at 10 μg/mL for 72 h. Then, the target MEC-1 cell line was stained with 10 µM calcein-AM (Biolegend) following the manufacturer´s instructions and co-cultured with PBMCs at a 25:1 (effector: target) ratio for 4 h. Calcein release was measured on a Varioskan™ LUX multimode microplate reader. For ADCC assays, MEC-1 cell line was pre-treated with 10 μg/mL rituximab or control IgG for 30 min before co-culturing.

### 2.6. Determination of Soluble BTLA on the Sera of Patients with CLL

Levels of soluble BTLA (sBTLA) in the sera from untreated patients and HD were determined by ELISA assay following the manufacturer´s protocol (Cusabio, Wuhan, China).

### 2.7. In Silico Analysis of the Role of BTLA in CLL and other Hematological Malignancies

BTLA and HVEM mRNA analyses were performed using transcriptome data available on Gene Expression Omnibus (GEO) repository using ShinyGeo (https://gdancik.shinyapps.io/shinyGEO/, accessed on 8 September 2020) (GEO22762, GSE22529, GSE5006, GSE31048 and GSE21029) [61]. A subset of 107 patients was employed to assess the impact of BTLA and HVEM expression on patients’ OS (GEO22762). BTLA and HVEM expression was evaluated in different solid tumors and hematological malignancies using RNAseq data from the TCGA database using GENT2 and GEPIA2 (http://gent2.appex.kr/gent2/ and http://gepia2.cancer-pku.cn/#general, accessed on 26 August 2021) [62,63].

### 2.8. Statistics

The relationship between continuous and categorical prognostic variables was evaluated by Mann–Whitney *U*-test, and Wilcoxon Matched-Pairs Signed Ranks test was used for intra-group comparisons. For time-to-event analysis, Kaplan–Meier curves were plotted and each group was compared by log-rank test. Relationship between patient characteristics and survival was evaluated by univariate Cox proportional hazards models using SPSS v23.0 software. *p* values of ≤ 0.05 were considered statistically significant. Adjusted *p*-values are calculated using the survMisc package as previously described [61].

## 3. Results

### 3.1. BTLA/HVEM Axis Is Dysregulated on Leukemic Cells and Impacts on Overall Survival

**Figure 1:**
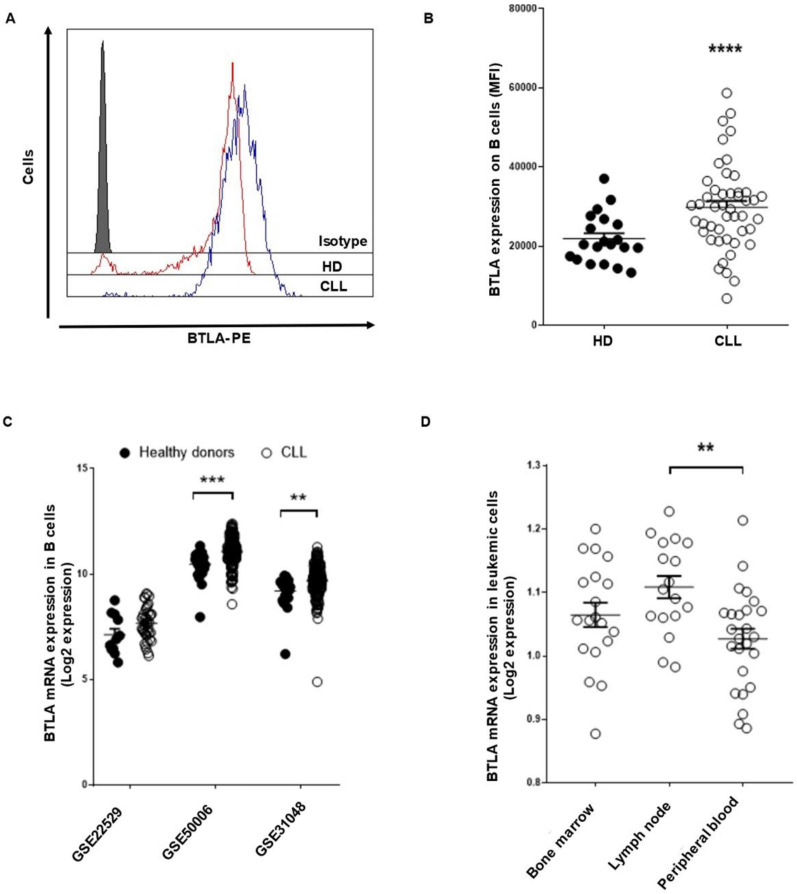
BTLA immune checkpoint expression is upregulated on leukemic cells from patients with CLL. BTLA surface expression on leukemic cells from patients with CLL (*n* = 46) and B cells from HD (*n* = 20) was evaluated by flow cytometry. (**A**) Representative histogram of BTLA expression (MFI) in HD and patients. (**B**) Comparison of BTLA levels between leukemic cells from patients and B cells from controls (MFI ± SEM). (**C**) Three available microarray data from the GEO database were interrogated to analyze *BTLA* mRNA expression in leukemic cells from patients with CLL in comparison with HD. (**D**) *BTLA* mRNA levels were evaluated according to leukemic cell localization (GSE21029). Each dot represents an individual sample. ** *p* < 0.01, *** *p* < 0.001 and **** *p* < 0.0001.

Surface expression of BTLA and HVEM was evaluated in 46 consecutive non-treated patients with CLL (Table 1) and 20 HD using flow cytometry. BTLA expression was significantly increased on leukemic cells from patients with CLL compared to their healthy counterpart [mean fluorescence intensity (MFI) ± standard error of mean (SEM): 30,363.3 ± 1593 vs. 21,905 ± 1394, *p* < 0.0001] (Figure 1A,B). The upregulation of BTLA expression in patients with CLL was validated by in silico analysis of RNAseq data from publicly available GEO datasets. *BTLA* mRNA expression was increased in the 3 datasets studied (GSE2259 *p* = n.s., GSE50006 *p* < 0.0001 and GSE31048, *p* = 0.0012) (Figure 1C). Of note, GSE21029 analysis revealed that leukemic cells from lymph nodes display higher *BTLA* mRNA expression than peripheral blood leukemic cells (*p* = 0.003) (Figure 1D).

**Figure 2:**
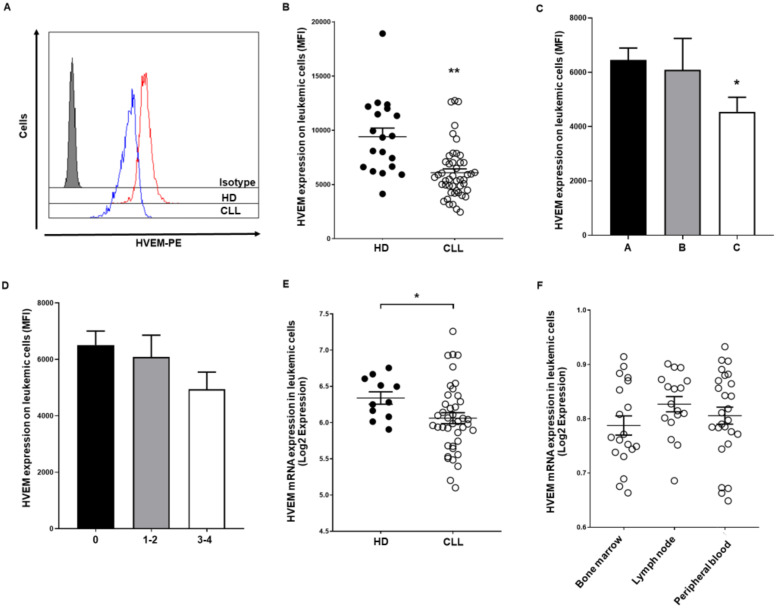
HVEM surface expression is decreased on leukemic cells from patients with CLL. HVEM expression was evaluated on leukemic cells from patients with CLL (*n* = 46) and B cells from HD (*n* = 20) by flow cytometry. Representative histogram (**A**) and comparison (**B**) of HVEM expression on leukemic and B cells from patients with CLL and controls (MFI ± SEM) are shown. Comparison of HVEM surface expression in patients stratified by Rai stage (**C**) and Binet stage (**D**) (MFI ± SEM) are depicted. (**E**) Microarray data from the GEO database were employed to determine *HVEM* mRNA expression in patients with CLL regarding HD (GSE22529). (**F**) *HVEM* mRNA levels were evaluated according to leukemic cell localization (GSE21029). * *p* < 0.05, ** *p* < 0.01.

HVEM expression was significantly downregulated on leukemic cells from patients with CLL (MFI: 6091 ± 358.2 vs. 9407 ± 795.2, *p* = 0.0015) (Figure 2A,B). Clinical analysis unveiled that HVEM, but not BTLA expression, is decreased in advanced Rai-Binet stage patients (Figure 2C,D). More concisely, patients with Binet stage C (*p* < 0.05) and Rai stage 3−4 stage (*p* = n.s.) exhibited decreased HVEM surface expression. No correlation between BTLA or HVEM expression levels and *IGHV* mutational status or cytogenetic alterations were observed in our cohort or in silico analysis, suggesting that BTLA/HVEM expression is independent of the most common prognostic factors in CLL. In silico analysis of previously published RNAseq studies revealed that *HVEM* mRNA is decreased in CLL compared to HD in all datasets, although it only achieved statistical significance in GSE22529 dataset (*p* = 0.03) (Figure 2E). No differences were observed regarding HVEM expression and leukemic cell location (Figure 2F). 

BTLA and HVEM expression were next evaluated in other hematological malignancies using publicly available datasets by means of GEPIA2 and GENT2 tools. GEPIA2 analysis agreed with previous studies reporting increased expression of BTLA on DLBCL (Figures S1 and S2) [42]. Of note, similar high *BTLA*/*HVEM* mRNA expression levels were observed in CLL, acute myeloid leukemia (AML), multiple myeloma (MM) and DLBCL, suggesting that BTLA/HVEM axis may also play a role in the pathogenesis of these malignancies (Figure S3). 

Since BTLA is not the sole binding partner for HVEM, CD160 and LIGHT expression were analyzed in patients with CLL (*n* = 41) (Figure S4). Our data agree with previous reports relative to increased expression of CD160 on leukemic cells (Figure S4A), whereas no difference on LIGHT surface expression was detected (Figure S4B) [52,53,64]. No differences regarding CD160 or LIGHT expression were observed on NK cells from patients with CLL (Figure S4B,C).

**Figure 3:**
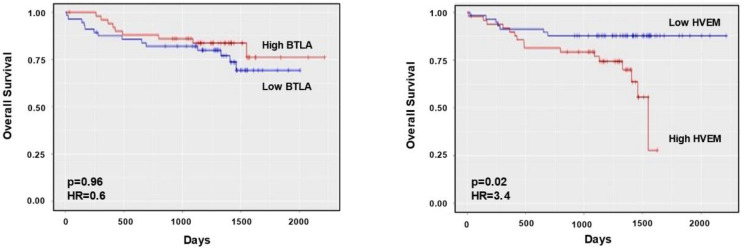
Impact of HVEM and BTLA on overall survival of patients with CLL. The relevance of *BTLA* (**A**) and *HVEM* (**B**) mRNA expression categorized by their mRNA levels in survival (GSE22762, *n* = 107) was assessed by a Kaplan–Meier survival analysis using ShinyGeo tool.

The impact of *HVEM* and *BTLA* mRNA expression on the survival of 107 patients with CLL (GSE22762) was assessed by a Kaplan–Meier survival analysis using ShinyGeo tool (Figure 3). Despite the fact that *BTLA* mRNA expression did not directly affect OS (HR = 0.6, *p* (adjusted) = 0.96), high *HVEM* expression correlated with poor OS (HR = 3.4, *p* (adjusted) = 0.02). Contrarily, *CD160* gene expression correlated with better prognosis (HR = 0.16, *p* (adjusted) = 0.001), whereas no influence of *LIGHT* expression was observed (Figure S4). Altogether, these data indicate that the BTLA/HVEM axis is dysregulated and significantly impacts OS of patients with CLL.

### 3.2. Soluble BTLA Levels Are Increased and Correlate with Time to Treatment in CLL

**Figure 4:**
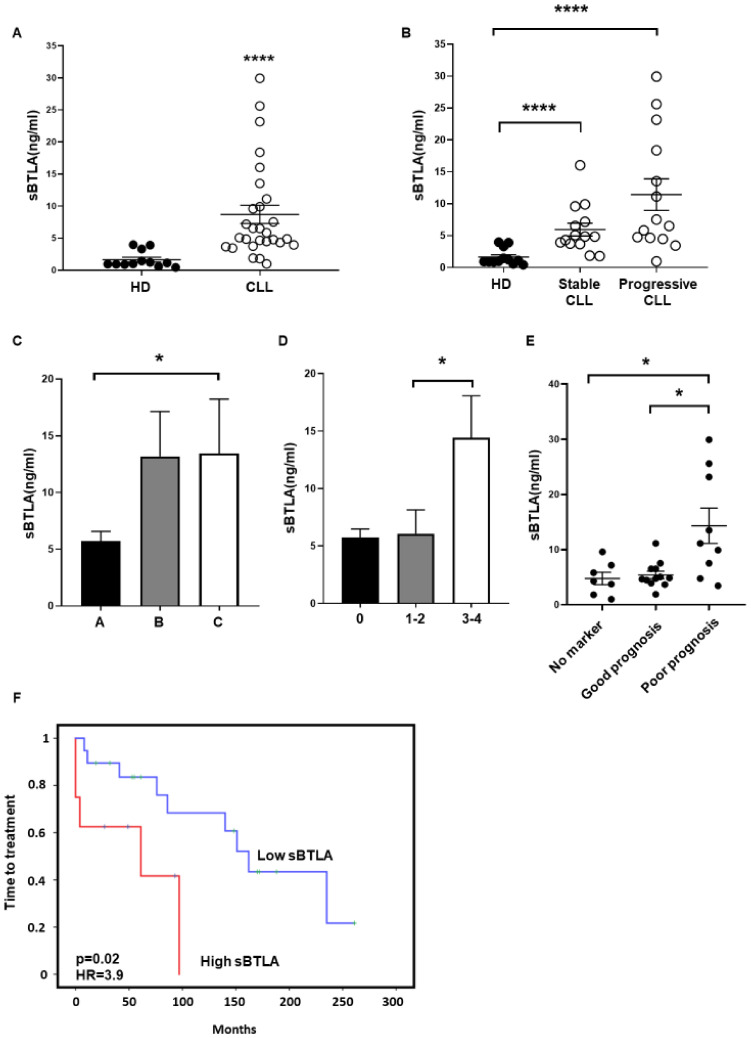
Soluble BTLA is increased in patients with CLL and affects time to treatment. (**A**) Sera levels of BTLA (sBTLA) were evaluated in 28 patients and 12 HD by ELISA. (**B**) sBTLA levels (ng/mL) in patients with CLL stratified by progression are represented. (**C**,**D**) Comparison of sBTLA (ng/mL) in patients stratified by Binet (**C**) and Rai (**D**) stages. (**E**) sBTLA levels in patients stratified by cytogenetic abnormalities: no marker (no cytogenetic abnormalities), good prognosis (patients with only del(13q)) and poor prognosis (patients with del(11q), del(17p) or trisomy 12, owing or not to del(13q)). (**F**) Kaplan–Meier survival analysis showing time to treatment relative to sBTLA sera levels from patients with CLL. * *p* < 0.05, **** *p* < 0.0001.

ELISA analysis of sera obtained from 28 patients with CLL and 12 HD showed a sharp augment of sBTLA in patients compared to HD (8.6 ± 1.4 ng/mL vs. 1.6 ± 0.3 ng/mL, *p* < 0.0001) (Figure 4A). Interestingly, patients with stable disease (stable CLL) presented lower levels of sBTLA than those who progressed and required therapeutic intervention (progressive CLL), although it did not reach statistical significance (Figure 4B). Moreover, higher levels of sBTLA were observed in advanced Rai versus 0 stage (5.7 ± 0.7 ng/mL vs. 14.4 ± 3.6 ng/mL, *p* = 0.07 and 6.0 ± 2.0 ng/mL vs. 14.4 ± 3.6 ng/ml, *p* = 0.02, respectively) and Binet C stage patients (5.7 ± 0.8 ng/ml vs. 13.1 ± 3.9 ng/mL, *p* = 0.05 and 13.4 ± 4.0 ng/mL vs. 13.1 ± 3.9 ng/mL, *p* = n.s.) (Figure 4C,D). Patients carrying cytogenetic alterations associated with poor outcome (del(11q), del(17p) or trisomy 12, owing or not to del(13q)) exhibited higher levels of sBTLA than patients with no cytogenetic abnormalities (4.7 ± 1.1 ng/mL vs. 14.3 ± 3.1 ng/mL, *p* = 0.01) and those ones with del(13q) (5.4 ± 0.6 ng/mL vs. 14.3 ± 3.1 ng/mL, *p* = 0.01) (Figure 4E), unraveling that higher levels of sBTLA are associated with advanced and aggressive CLL. In agreement, Kaplan–Meier analysis displayed that increased sBTLA was significantly associated with diminished TTT (*p* = 0.02, HR = 3.9) (Figure 4F).

### 3.3. BTLA Is Upregulated on NK Cells and Is Associated with Poor Outcome

**Figure 5:**
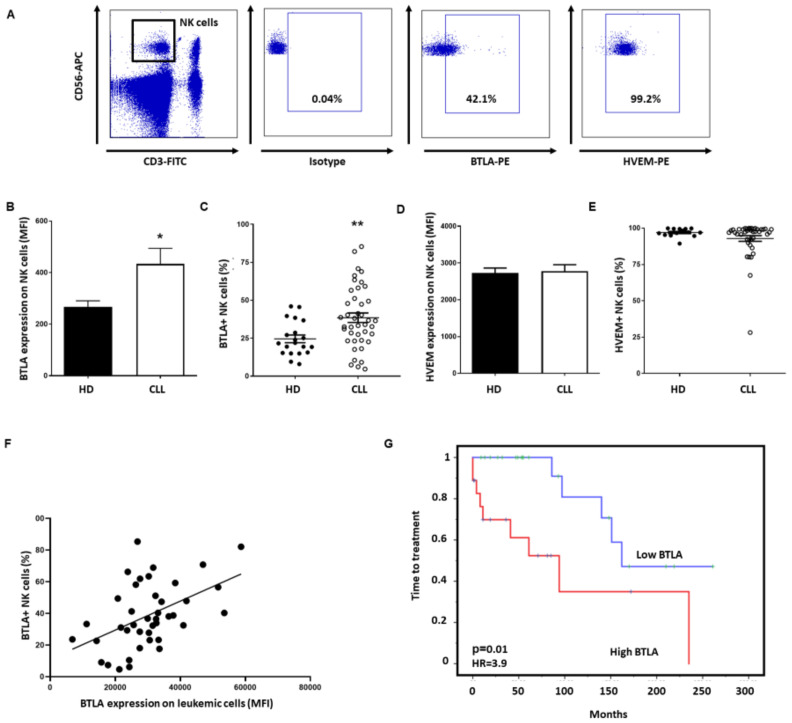
BTLA expression is increased on NK cells and impacts time to treatment. (**A**) Dot plot showing the gating strategy for NK cell identification and evaluation of BTLA+ and HVEM+ populations. (**B**) Comparison of BTLA surface expression between patients with CLL (*n* = 42) and HD (*n* = 20) (MFI ± SEM). (**C**) Percentage of BTLA+ NK cells in patients with CLL compared to HD. (**D**,**E**) HVEM surface expression (**D**) and the percentage of HVEM+ NK cells (**E**) in patients with CLL compared to HD. (**F**) Correlation between the percentage of NK cells expressing BTLA and its expression on leukemic cells (*p* = 0.0015). (**G**) Kaplan–Meier survival analysis showing TTT in patients with CLL categorized by BTLA levels. * *p* < 0.05, ** *p* < 0.01.

To analyze whether BTLA/HVEM axis may exert an immunosuppressive effect on NK cells, their expression on these immune subsets was next analyzed by flow cytometry (Figure 5A). BTLA expression on NK cells (MFI: 417.6 ± 57.0 vs. 266.5 ± 24.0, *p* = 0.05) (Figure 5B) and the percentage of BTLA+ NK cells was significantly increased in patients (38.4% ± 3.1% vs. 24.5 ± 2.5, *p* = 0.005) (Figure 5C), but no difference in HVEM expression on NK cells was observed (Figure 5D,E). Noticeably, BTLA expression on leukemic cells significantly correlated with the percentage of BTLA+ NK cells (Pearson´s rank test *p* = 0.0015) (Figure 5F). Besides, Kaplan–Meier analysis demonstrated that high expression of BTLA on NK cells from patients with CLL is associated with shorter TTT (*p* = 0.01, HR = 3.9) (Figure 5G). Accordingly, BTLA overexpression on NK cells may directly affect outcome in patients with CLL.

### 3.4. BTLA Decreases IFN-γ Production and NK Cell-Mediated Cytotoxicity, But It May Be Reversed by BTLA Blockade 

**Figure 6:**
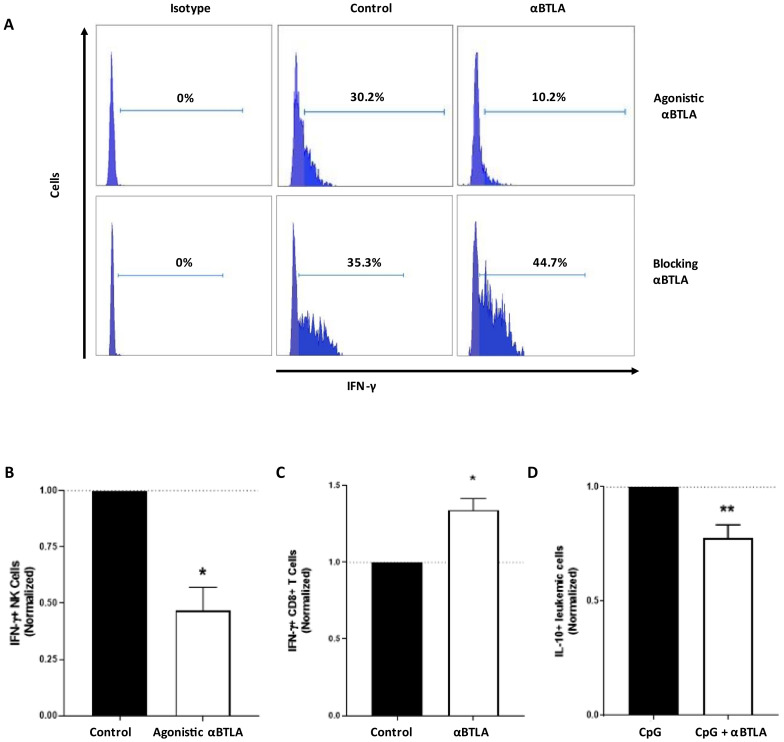
BTLA induces NK cell immunosuppression, but it may be reverted by BTLA/HVEM axis disruption. (**A**) Representative flow cytometry histograms of IFN-γ+ NK cells from patients with CLL treated with agonistic or blocking anti-BTLA mAb. (**B**) PBMCs from patients with CLL were cultured with plate-coated agonistic anti-BTLA or control IgG (10 µg/mL) for 24 h (*n* = 6) and IFN-γ+ NK cells were assessed by flow cytometry (normalized to control). (**C**) PBMCs from patients were treated with blocking anti-BTLA or isotype-matched IgG (10 µg/mL) for 72 h and IFN-γ+ NK cells were evaluated by flow cytometry (*n* = 7) (normalized to control). (**D**) PBMCs from patients with CLL were treated with CpG (200 ng/mL) alone or in combination with blocking anti-BTLA or control IgG (10 µg/mL) for 72 h and IL-10+ leukemic cells were evaluated by flow cytometry (*n* = 8) (normalized to CpG-treated PBMCs). * *p* < 0.05, ** *p* < 0.01.

The functional consequences of BTLA expression on NK cells were assessed upon treatment with agonistic and antagonistic mAbs. Decreased IFN-γ production by NK cells was observed after treating with an agonistic anti-BTLA mAb suggesting an inhibitory role for BTLA on NK cell activity (35.6 ± 8.9 vs. 20.8 ± 10.2, *p* = 0.03) (Figure 6). Contrarily, BTLA/HVEM signaling disruption with a blocking mAb significantly augmented percentage of IFN-γ+ NK cells (35.3 ± 9.3 vs. 44.7 ± 9.8, *p* = 0.01). In agreement with previous reports, BTLA blockade significantly decreased the production of the immunosuppressive cytokine IL-10 by leukemic cells (35.2 ± 9.2 vs. 30.0 ± 9.2, *p* = 0.007) [48]. Taken together, these results suggest that BTLA plays a role in promoting NK cell-mediated immunosuppression in CLL, which may be partially revoked by BTLA/HVEM disruption.

**Figure 7:**
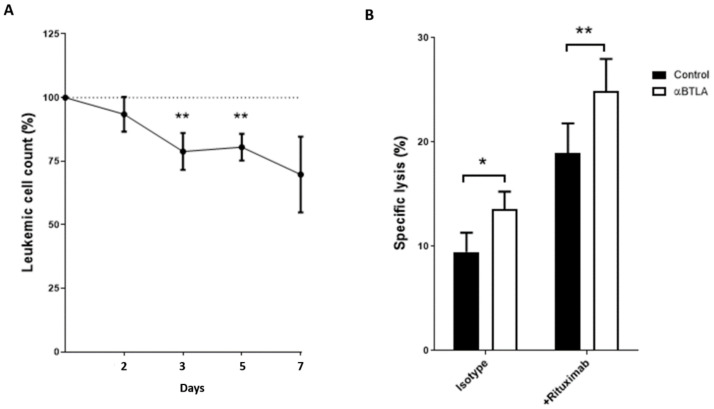
BTLA blockade depletes leukemic cells and increases NK cell-mediated cytotoxicity. (**A**) PBMCs from patients with CLL were treated with blocking anti-BTLA mAb or control IgG (10 µg/mL) and absolute leukemic cell count was evaluated at indicated timepoints. (**B**) The effect of BTLA blockade on NK cell-mediated cytotoxicity was evaluated by calcein-AM assay. PBMCs from patients were treated with blocking anti-BTLA mAb or control IgG (10 µg/mL) (*n* = 11) for 72 h and then co-cultured with MEC-1 cell line (pre-treated with 10 µg/mL of rituximab or control IgG for 30 min) at 25:1 (effector: target) ratio for 4h. * *p* < 0.05, ** *p* < 0.01.

Noteworthy, treatment of PBMCs from patients with CLL with anti-BTLA blocking mAb significantly depleted leukemic cell numbers at 48 and 72 h compared with control IgG (Figure 7A). In consonance with such depletion of leukemic cells, BTLA blockade significantly increased NK cell-mediated cytotoxicity in co-culture experiments employing the CLL-derived cell line MEC-1 as target cells. These results suggest that leukemic depletion may be due, at least in part, to increased NK cell antitumor activity (Figure 7B). In the same line, pre-treatment of target cells with 10 µg/mL of rituximab significantly enhanced ADCC activity in the presence of BTLA mAb (Figure 7B). Overall, our data bring to light that BTLA blockade, alone or in combination with rituximab, has a significant effect on the elimination of leukemic cells that is associated with enhanced NK cell-mediated cytotoxicity. 

## 4. Discussion

The landscape of available therapies for CLL management has been enriched by the inclusion of small-molecules inhibitors, such as ibrutinib, idelalisib or venetoclax [5,6,7]. However, resistance mechanisms related to these novel treatments, together with the profound immunosuppression associated to CLL progression suggest that ICB-based therapy could benefit a subset of these patients. 

BTLA is an inhibitory checkpoint protein that, upon engagement with HVEM, negatively regulates T cell-mediated responses. Interestingly, targeting BTLA was able to boost proliferation and cytokine production by T lymphocytes in melanoma in vivo and in vitro [49]. Moreover, BTLA blockade, alone or in combination with chemotherapy, improved survival in epithelial ovarian carcinoma murine models [48]. In hematological malignancies, BTLA/HVEM axis dysregulation decreased perforin and granzyme B production by BTLA^+^ T cells and was associated with poor outcome in DLBCL and FL, whereas only two studies reported about BTLA and HVEM expression on leukemic cells from CLL [42,51,55]. Altogether, along with the recent approval of one anti-BTLA mAb by FDA (TAB004/JS004, Junshi Biosciences, Shanghai, China) have attracted attention at this novel checkpoint. However, scarce data are available about the role of BTLA and its ligand on NK cells.

Herein, we highlight the potential of BTLA as a therapeutic target in CLL, unveiling that BTLA expression is highly upregulated in patients with CLL, whereas HVEM expression is downregulated. Higher *BTLA* mRNA expression in leukemic cells from lymph nodes was observed in comparison to peripheral blood, fitting with previously described expression patterns in proliferation centers in CLL [56]. However, the rationale behind such increased expression of BTLA, an inhibitory receptor, on leukemic cells remains elusive [55,65]. Whether this expression pattern is the result of the continuous activation status of leukemic cells or an inefficient autocrine inhibitory loop, and its possible implication in CLL pathogenesis deserves further investigations [55]. 

Regarding HVEM, reduced expression was observed in patients with advanced Binet stage. Similar to FL, increased HVEM expression correlated with poorer OS in CLL. In FL, transformation to DLBCL and lower OS was associated to diminished BTLA expression and augmented HVEM expression on tumor cells [51]. The poorer outcome observed in high HVEM-expressing patients with FL may be related to HVEM function as a signal-transducing receptor, thus activating NF-κB and AKT pathways that may promote proliferation and survival of leukemic cells [66]. 

In consonance with leukemic cells, NK cells also showed an augmented surface expression of BTLA, and high BTLA expression on NK cells significantly correlated with reduced TTT, suggesting that this checkpoint may play an important immunosuppressive role in CLL. HVEM is considered a “molecular switch” due to its ability to interact with either co-stimulatory or co-inhibitory receptors, providing the rationale for a dual role of BTLA/HVEM axis in CLL. On one hand, increased expression of BTLA may trigger inhibitory signaling on NK cells. On the other hand, downregulation of HVEM on leukemic cells may lead to decreased activating signaling through co-stimulatory molecules, such as LIGHT. Altogether, this dysregulation may lead to immunosuppression and functional inhibition of NK cells [67]. Concordantly, diminished IFN-γ production after treatment with agonistic anti-BTLA mAb was observed, whereas treatment with blocking anti-BTLA mAb (Genentech) promoted significant antitumor activities. BTLA blockade induced leukemic cell depletion and promoted NK cell-mediated responses. Further, as previously reported, BTLA blockade decreased the production of the immunosuppressive and tumor-promoting cytokine IL-10 by B cells, suggesting that targeting BTLA signaling may restore, at least in part, NK cell immune competence [48,68,69,70]. Importantly, a recent work has showed that inhibition of IL-10 boosts ICB-related anti-tumor responses in CLL [71]. In line with this, BTLA blockade promoted NK cell-mediated cytotoxicity and rituximab-induced ADCC in CLL. Overall, BTLA/HVEM axis disruption may promote NK cell-mediated antitumor responses and our results provide the rationale for combining anti-BTLA therapy along with anti-CD20 mAbs. The aim of this work was to elucidate whether BTLA/HVEM axis may induce diminished NK cell-mediated anti-tumor responses. However, since BTLA and HVEM are highly expressed on T cells, whether BTLA blockade may promote T cell-mediate anti-tumor responses in patients with CLL deserves further investigations. 

In addition, we also found that sBTLA is a prognostic factor for outcome in patients with CLL. sBTLA was sharply increased in sera from patients with CLL, particularly in those with cytogenetic abnormalities classically associated with adverse outcome [72]. Interestingly, no detectable levels of sBTLA were obtained from leukemic cells cultured ex vivo, thus suggesting that tumor microenvironment may play a role in sBTLA production. Importantly, high sBTLA was associated with reduced TTT in CLL. Increased levels of sBTLA in sera from patients with cancer and its association with OS has previously been reported in hepatocellular carcinoma, gastric cancer, and pancreatic adenocarcinoma, although the underlying mechanism remains elusive [45,46,47,73]. The role of sBTLA has not yet been established. Nevertheless, sBTLA may promote an activating autocrine loop through its interaction with HVEM on leukemic cells. Additionally, as other soluble NK cell ligands, it may modulate anti-tumor responses by concomitantly impeding HVEM binding to the activating receptors on NK and T cells [74,75,76]. The underlying mechanism and the potential role of serum levels of sBTLA as a predictor factor for poor outcome in patients with CLL deserve further investigation.

## 5. Conclusions

In summary, we found that the BTLA/HVEM axis is deeply dysregulated in leukemic cells and NK cells from patients with CLL, leading to immunosuppression and diminished NK cell-mediated immunosurveillance. This dysregulation is associated with poor outcome, unveiling a role of BTLA/HVEM in the pathogenesis of CLL. Further, BTLA blockade restored, at least in part, the immunosuppression of NK cells, suggesting that targeting BTLA may be a potential therapeutic strategy to be explored in this disease and other hematological malignancies.

## Figures and Tables

**Table 1:** Clinical characteristics of patients with chronic lymphocytic leukemia.

Patients	Patients (*n* = 46)	%
Age		
Years (mean)	72.9	
Sex		
Female	21	45.6
Male	25	54.3
Rai Stage		
0	24	54.5
I–II	14	27.2
III–IV	8	18.1
Binet Stage		
A	34	73.9
B	35	10.8
C	7	15.2
Cytogenetic abnormalities (FISH)		
No alterations	11	23.9
del(13q)	19	41.3
del(11q)	4	8.6
del(17p)	5	10.8
Trisomy 12	5	10.8
Others	1	2.1
Complex karyotype	6	13
*IGHV* status		
Mutated	36	78.2
Unmutated	10	21.7
Progression		
Stable disease	29	63
Progressive disease	17	36.9

## Data Availability

In silico analysis were performed from publicly available datasets from Gene Expression Omnibus (GEO) repository using ShinyGeo (https://gdancik.shinyapps.io/shinyGEO/) (GEO22762, GSE22529, GSE5006, GSE31048 and GSE21029) and RNAseq data from the TCGA database using GENT2 and GEPIA2 (http://gent2.appex.kr/gent2/ and http://gepia2.cancer-pku.cn/#general).

## Supplementary Materials

The following are available online at https://www.mdpi.com/article/10.3390/cancers13081766/s1, Figure S1: BTLA expression is dysregulated in a wide variety of cancers including DLBCL and AML, Figure S2: HVEM expression is upregulated in LAML, Figure S3: BTLA and HVEM expression in different hematological malignancies, Figure S4: CD160 and LIGHT expression in patients with CLL and correlation with OS.
