# Supplementary material for: BTLA/HVEM Axis Induces NK Cell Immunosuppression and Poor Outcome in Chronic Lymphocytic Leukemia
Source: Cancers (Basel). 2021 Apr 7;13(8):1766. doi: 10.3390/cancers13081766 (PMC8067870; doi:10.3390/cancers13081766)
Supplement: Supplementary file 1 [file cancers-13-01766-s001.pdf]

Supplementary Materials:

BTLA/HVEM Axis Induces NK Cell Immunosuppression and Poor Outcome in Chronic Lymphocytic Leukemia

Christian Sordo-Bahamonde [1,2,3], Seila Lorenzo-Herrero [1,2,3], Ana P Gonzalez-Rodriguez [2,3,4,*], Angel R. Payer [2,3,4], Esther González-García [5], Alejandro López-Soto [2,3,6] and Segundo Gonzalez [1,2,3,*]

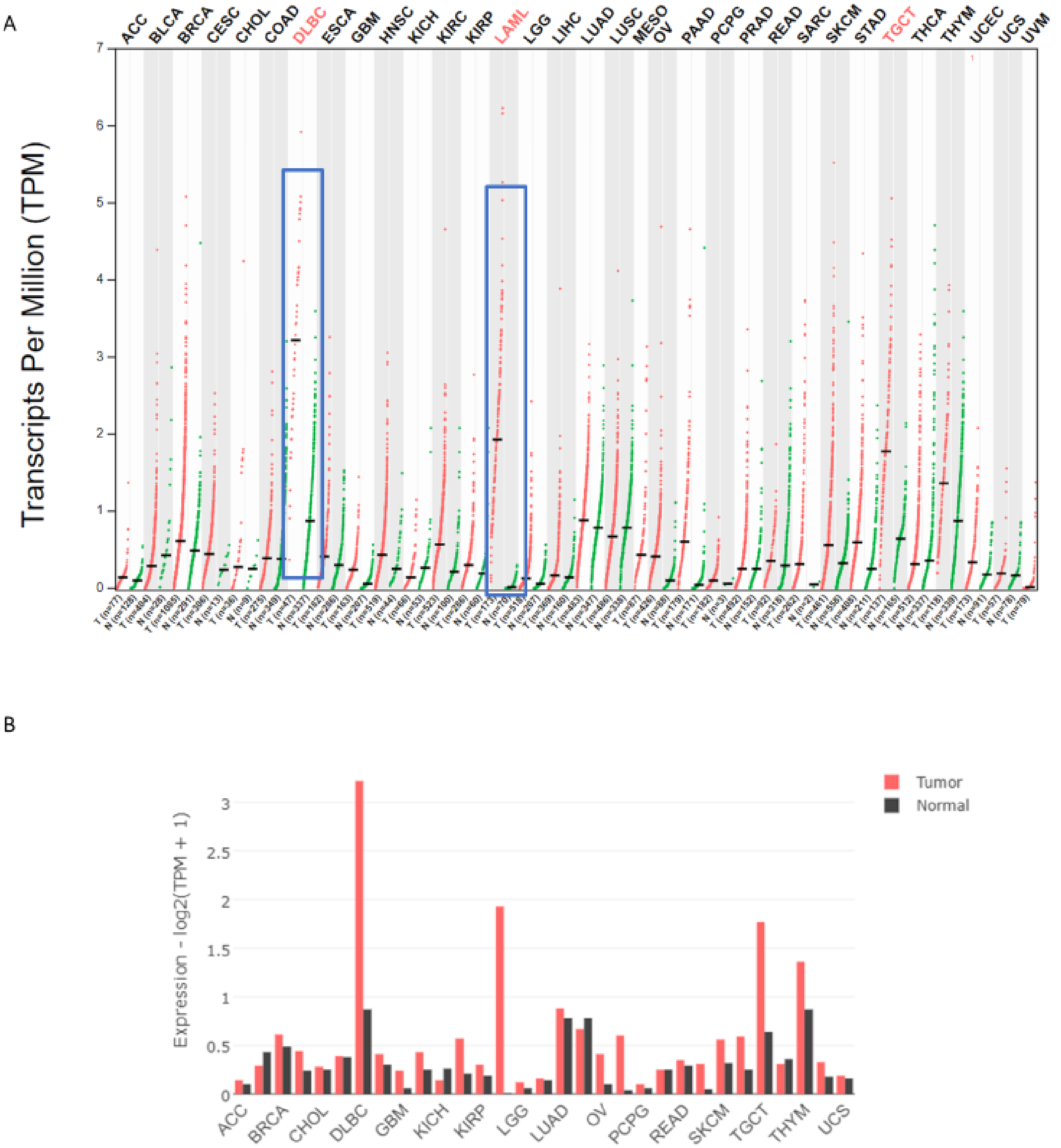

Figure 1S. *BTLA* expression is dysregulated in a wide variety of cancers including DLBCL and AML. *BTLA* mRNA expression was evaluated in publicly available datasets by GEPIA2 (A) and GENT2 (B) tools. Abbreviations: ACC: Adrenocortical carcinoma; BLCA: Bladder Urothelial Carcinoma; BRCA: Breast invasive carcinoma; CESC: Cervical squamous cell carcinoma and endocervical adenocarcinoma; CHOL: Cholangio carcinoma; COAD: Colon adenocarcinoma; DLBC: Lymphoid Neoplasm Diffuse Large B-cell Lymphoma; ESCA: Esophageal carcinoma; GBM: Glioblastoma multiforme; HNSC: Head and Neck squamous cell carcinoma; KICH: Kidney Chromophobe; KIRC: Kidney renal clear cell carcinoma; KIRP: Kidney renal papillary cell carcinoma; LAML: Acute Myeloid Leukemia; LGG: Brain Lower Grade Glioma; LIHC: Liver hepatocellular carcinoma; LUAD: Lung adenocarcinoma; LUSC: Lung squamous cell carcinoma; MESO: Mesothelioma; OV: Ovarian serous cystadenocarcinoma; PAAD: Pancreatic adenocarcinoma; PCPG: Pheochromocytoma and Paraganglioma; PRAD: Prostate adenocarcinoma; READ: Rectum

adenocarcinoma; SARC: Sarcoma; SKCM: Skin Cutaneous Melanoma; STAD: Stomach adenocarcinoma; TGCT: Testicular Germ Cell Tumors; THCA: Thyroid carcinoma; THYM: Thymoma; UCEC: Uterine Corpus Endometrial Carcinoma; UCS: Uterine Carcinosarcoma; UVM: Uveal Melanoma

A

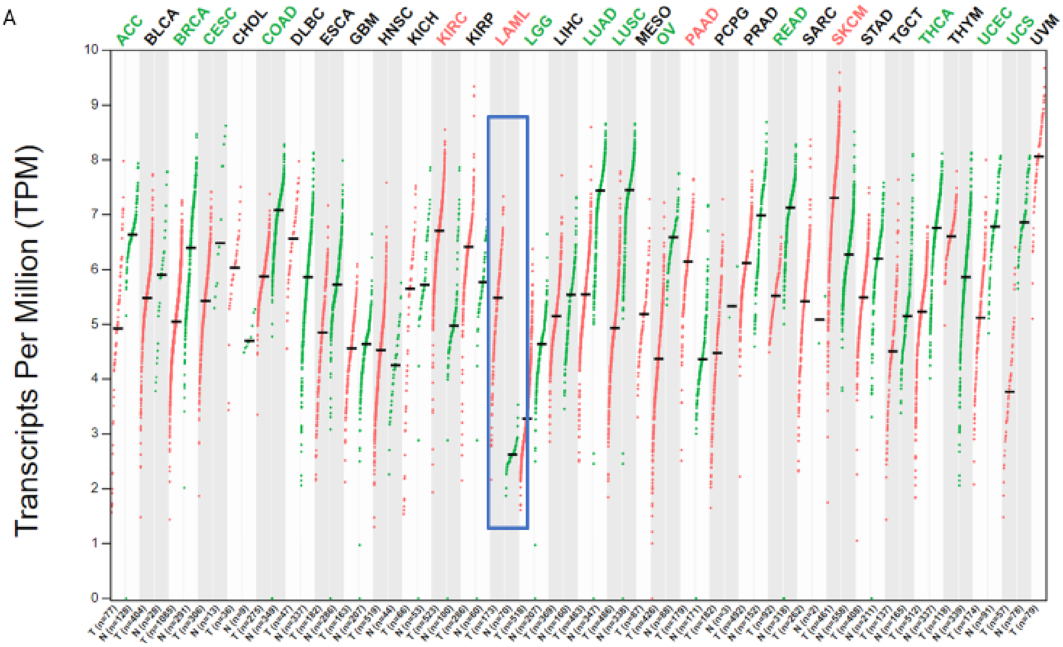

B

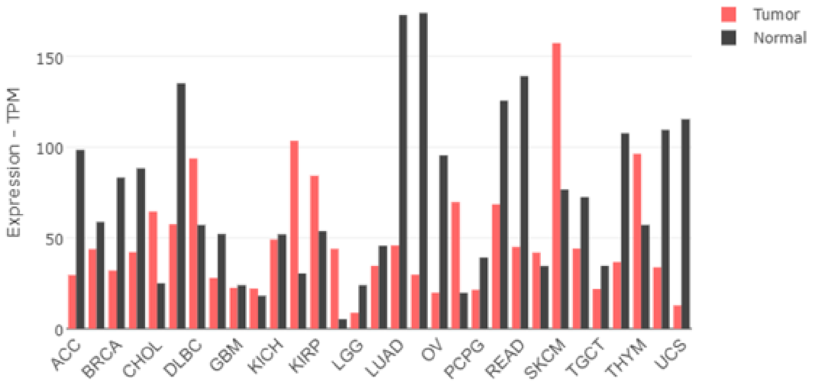

Figure 2S. *HVEM* expression is upregulated in LAML. Publicly available data were analyzed by GEPIA2 (A) and GENT2 (B) tools. Abbreviations: ACC: Adrenocortical carcinoma; BLCA: Bladder Urothelial Carcinoma; BRCA: Breast invasive carcinoma; CESC: Cervical squamous cell carcinoma and endocervical adenocarcinoma; CHOL: Cholangio carcinoma; COAD: Colon adenocarcinoma; DLBC: Lymphoid Neoplasm Diffuse Large B-cell Lymphoma; ESCA: Esophageal carcinoma; GBM: Glioblastoma multiforme; HNSC: Head and Neck squamous cell carcinoma; KICH: Kidney Chromophobe; KIRC: Kidney renal clear cell carcinoma; KIRP: Kidney renal papillary cell carcinoma; LAML: Acute Myeloid Leukemia; LGG: Brain Lower Grade Glioma; LIHC: Liver hepatocellular carcinoma; LUAD: Lung adenocarcinoma; LUSC: Lung squamous cell carcinoma; MESO: Mesothelioma; OV: Ovarian serous cystadenocarcinoma; PAAD: Pancreatic adenocarcinoma; PCPG: Pheochromocytoma and Paraganglioma; PRAD: Prostate adenocarcinoma; READ: Rectum adenocarcinoma; SARC: Sarcoma; SKCM: Skin Cutaneous Melanoma; STAD: Stomach adenocarcinoma; TGCT: Testicular Germ Cell Tumors; THCA: Thyroid carcinoma; THYM: Thymoma; UCEC: Uterine Corpus Endometrial Carcinoma; UCS: Uterine Carcinosarcoma; UVM: Uveal Melanoma

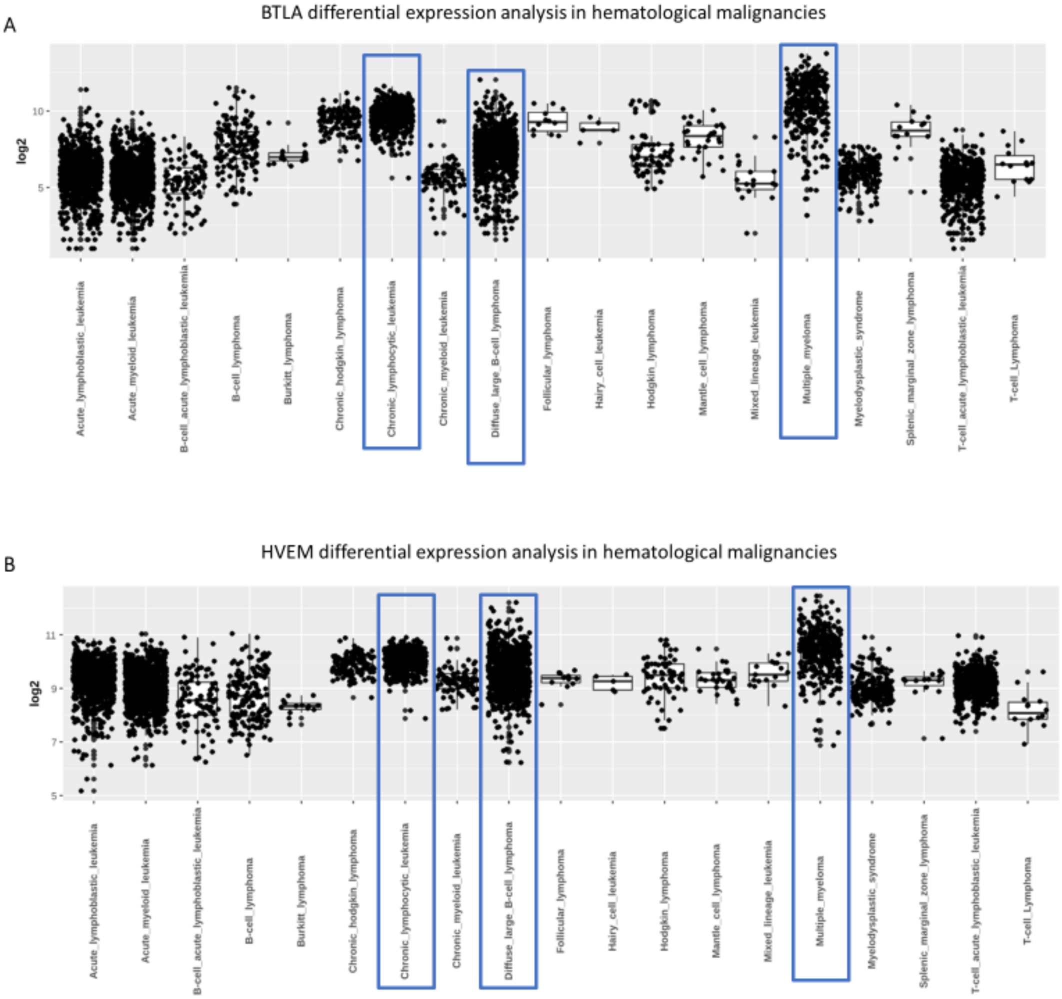

Figure 3S. BTLA and HVEM expression in different hematological malignancies. mRNA expression levels of *BTLA* (A) and *HVEM* (B) in hematological malignancies using GENT2 are represented.

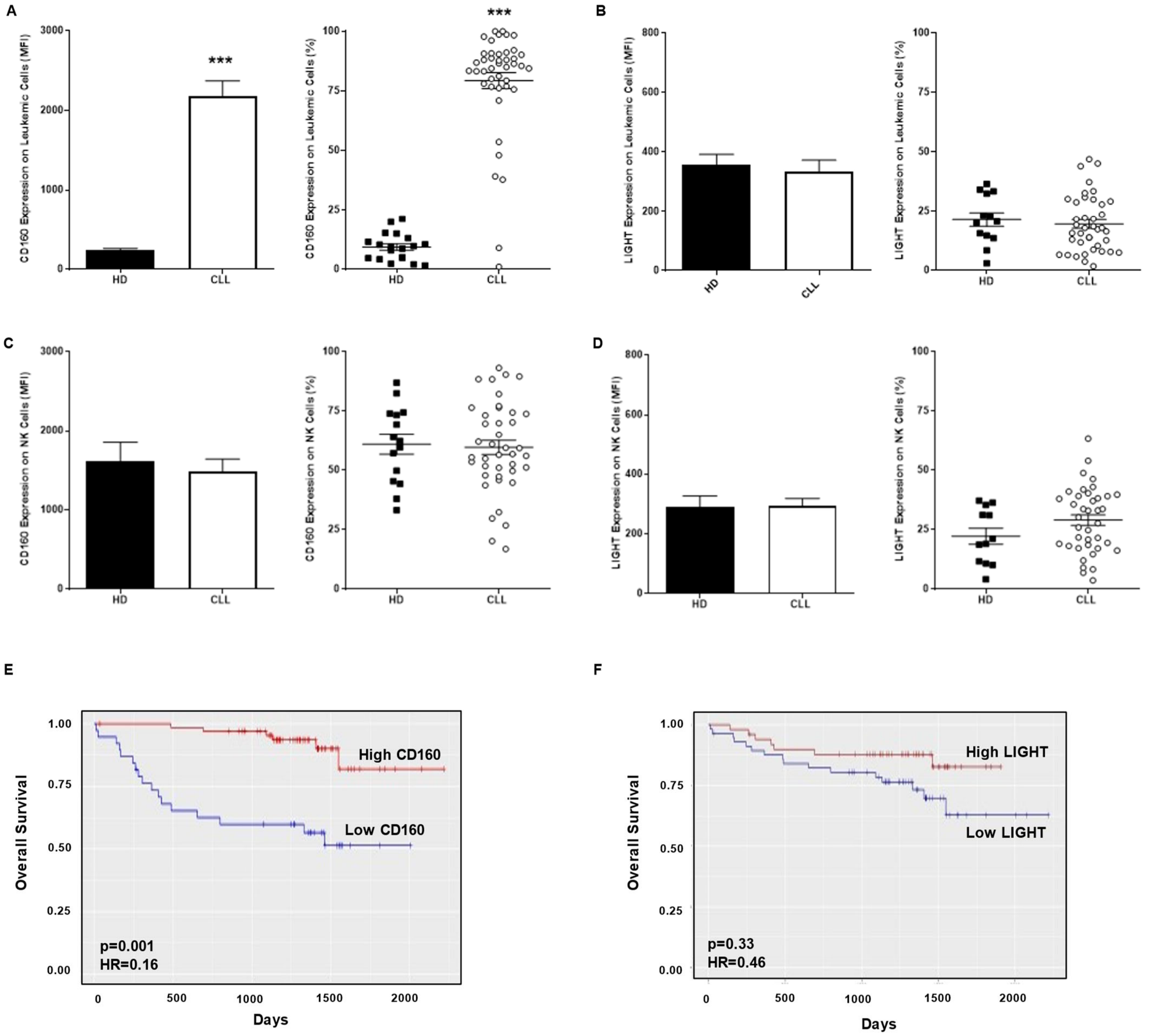

Figure 4S. CD160 and LIGHT expression in patients with CLL and correlation with OS. Surface expression of CD160 was evaluated in patients with CLL (n = 41) and HD (n = 12–18) by flow cytometry. Comparison between CD160 (A) and LIGHT (B) in leukemic cells and their healthy counterpart is shown. Expression levels of CD160 (C) and LIGHT (D) was assessed and compared in NK cells from HD and patients with CLL. MFI ± SEM and percentage of CD160/LIGHT positive cells are represented. Kaplan–Meier survival analysis relative to CD160 (E) and LIGHT (F) expression was performed using the GSE22762 dataset and ShinyGeo tool. ***p < 0.001. ***p < 0.001.
